# Effect of lung volume preservation during spontaneous breathing trial on successful extubation in patients receiving mechanical ventilation: protocol for a multicenter clinical trial

**DOI:** 10.1186/s13063-024-08297-1

**Published:** 2024-07-16

**Authors:** Carles Subirà, Gina Rognoni, Herbert Baquerizo, Carolina García, Sara Cabañes, Maria de la Torre, Beatriz Quevedo, Cristina Pedrós, Ana I. Tizón, Natalia Murillo, Laura Parro, Fernando Eiras, Gemma Rialp, Susana Altaba, Alejandro González-Castro, Andrés F. Pacheco, Pablo Bayoumi, Norma Gómez-Medrano, Imma Vallverdú, Áurea Higón, María D. Navarro, Alirio Falcón, Elena Keough, David Arizo, Juan F. Martínez, Núria Durán, Raquel Rodríguez, Melinda R. Popoviciu-Koborzan, Isabel Guerrero, Pablo Concha, Patricia Barral, Montserrat Batlle, Sílvia Cano, Silvia Garcia-Castrillon, Xavier Andorrà, Yenifher Tua, Anna Arnau, Rafael Fernández

**Affiliations:** 1 Servei de Medicina Intensiva, Hospital de La Santa Creu I Sant Pau, Barcelona, Spain; 2 Grup de Recerca en Malalt Crític (GMC), Institut de Recerca I Innovació en Ciències de La Vida I de La Salut a La Catalunya Central (IRIS-CC), Vic, Spain; 3 CIBER Enfermedades Respiratorias (CIBERES), Instituto de Salud Carlos III, 28029 Madrid, Spain; 4 Servei de Medicina Intensiva, Althaia Xarxa Assistencial Universitària de Manresa, Manresa, Spain; 5 Programa de Doctorat en Medicina I Ciències Biomèdiques, Universitat de Vic- Universitat Central de Catalunya (UVIC-UCC), Vic, Spain; 6 Servicio de Medicina Intensiva, Hospital Universitario de Canarias, San Cristóbal de La Laguna, Tenerife Spain; 7 Servicio de Medicina Intensiva, Txagorritxu Hospital Universitario Araba, Gasteiz, Spain; 8 Servei de Medicina Intensiva, Hospital de Mataró, Mataró, Spain; 9 Servicio de Medicina Intensiva, Hospital Clínico Universitario de Valencia, València, Spain; 10 Servei de Medicina Intensiva, Hospital General de Granollers, Granollers, Spain; 11 Servicio de Medicina Intensiva, Complexo Hospitalario Universitario de Ourense, Ourense, Spain; 12 Servei de Medicina Intensiva, Hospital Universitari de Tarragona Joan XXIII, Tarragona, Spain; 13 Servicio de Medicina Intensiva, Hospital Universitario del Henrares, Coslada, Spain; 14 Servicio de Medicina Intensiva, Hospital Universitario de Pontevedra, Pontevedra, Spain; 15 Servei de Medicina Intensiva, Hospital Son Llàtzer, Palma de Mallorca, Spain; 16 Servicio Medicina Intensiva, Hospital General Universitario de Castellón, Castelló de La Plana, Spain; 17 Servicio de Medicina Intensiva, Hospital Universitario Marqués de Valdecilla, Santander, Spain; 18 Servei de Medicina Intensiva, Hospital Universitari de La Vall d’Hebron, Barcelona, Spain; 19 Servicio de Medicina Intensiva, Hospital General Universitario Santa Lucía, Cartagena, Spain; 20 Servicio de Medicina Intensiva, Hospital General Universitario de Elche, Elx, Spain; 21 Servei de Medicina Intensiva, Hospital Universitari San Joan de Reus, Reus, Spain; 22 Servicio de Medicina Intensiva, Hospital General Universitario Morales Messeguer, Murcia, Spain; 23 Servicio de Medicina Intensiva, Hospital Arnau de Vilanova, Valencia, Spain; 24 Servei de Medicina Intensiva, Hospital Universitari Mútua de Terrassa, Terrassa, Spain; 25 Servicio de Medicina Intensiva, Hospital Universitario de La Princesa, Madrid, Spain; 26 Servicio de Medicina Intensiva, Hospital de Sagunto, Sagunt, Spain; 27 Servicio de Medicina Intensiva, Hospital Regional Universitario de Málaga, Malaga, Spain; 28 Servei de Medicina Intensiva, Hospital Universitari Sagrat Cor, Barcelona, Spain; 29 Servicio de Medicina Intensiva, Hospital Universitario Central de Asturias, Oviedo, Spain; 30 Instituto de Investigación Sanitaria del Principado de Asturias, Oviedo, Spain; 31 Servei de Medicina Intensiva, Hospital de Sant Joan Despí Moisès Broggi, Sant Joan Despí, Spain; 32 Servicio de Medicina Intensiva, Hospital Universitario Virgen de Las Nieves, Granada, Spain; 33 Servei de Medicina Intensiva, Hospital Verge de La Cinta, Tortosa, Spain; 34 Servicio de Medicina Intensiva, Complejo Hospitalario Universitario de Santiago, Santiago de Compostela, Spain; 35 Unitat de Recerca I Innovació, Althaia Xarxa Assistencial Universitària de Manresa, Manresa, Spain; 36 Grup de Recerca en Cronicitat de La Catalunya Central (C3RG), Institut de Recerca I Innovació en Ciències de La Vida I de La Salut a La Catalunya Central (IRIS-CC), Vic, Spain; 37 Facultat de Medicina, Universitat de Vic-Central de Catalunya (UVIC-UCC), Vic, Spain

**Keywords:** Mechanical ventilation, Weaning, Spontaneous breathing trial, Extubation, Lung ultrasound

## Abstract

**Background:**

In standard weaning from mechanical ventilation, a successful spontaneous breathing test (SBT) consisting of 30 min 8 cmH_2_O pressure-support ventilation (PSV8) without positive end-expiratory pressure (PEEP) is followed by extubation with continuous suctioning; however, these practices might promote derecruitment. Evidence supports the feasibility and safety of extubation without suctioning. Ultrasound can assess lung aeration and respiratory muscles. We hypothesize that weaning aiming to preserve lung volume can yield higher rates of successful extubation.

**Methods:**

This multicenter superiority trial will randomly assign eligible patients to receive either standard weaning [SBT: 30-min PSV8 without PEEP followed by extubation with continuous suctioning] or lung-volume-preservation weaning [SBT: 30-min PSV8 + 5 cmH_2_O PEEP followed by extubation with positive pressure without suctioning]. We will compare the rates of successful extubation and reintubation, ICU and hospital stays, and ultrasound measurements of the volume of aerated lung (modified lung ultrasound score), diaphragm and intercostal muscle thickness, and thickening fraction before and after successful or failed SBT. Patients will be followed for 90 days after randomization.

**Discussion:**

We aim to recruit a large sample of representative patients (*N* = 1600). Our study cannot elucidate the specific effects of PEEP during SBT and of positive pressure during extubation; the results will show the joint effects derived from the synergy of these two factors. Although universal ultrasound monitoring of lungs, diaphragm, and intercostal muscles throughout weaning is unfeasible, if derecruitment is a major cause of weaning failure, ultrasound may help clinicians decide about extubation in high-risk and borderline patients.

**Trial registration:**

The Research Ethics Committee (CEIm) of the Fundació Unió Catalana d’Hospitals approved the study (CEI 22/67 and 23/26). Registered at ClinicalTrials.gov in August 2023. Identifier: NCT05526053.

**Supplementary Information:**

The online version contains supplementary material available at 10.1186/s13063-024-08297-1.

## Introduction

### Background and rationale {6a}

Mechanical ventilation (MV) is the main reason for admission to intensive care units (ICU) [1]. Weaning refers to the entire process aimed at liberating the patient from the ventilator [2]. This process begins with screening patients for readiness to start weaning followed by a spontaneous breathing trial (SBT) to evaluate the patient’s ability to breath without the ventilator and extubation (i.e., the removal of the endotracheal tube); it can also include prophylactic treatments before or after extubation to prevent the need to reconnect MV [3–5]. Weaning failure and the reintubation are associated with bad prognosis, more infections, higher costs, and greater mortality [6]. Lungs can collapse during weaning not only when switching the ventilator from controlled-assisted modes to pressure-support ventilation (PSV) for an SBT but also during the extubation maneuver while suctioning. Weaning failure and reintubation are multifactorial, including airway or pulmonary dysfunction, decreased lung compliance, muscular weakness, and cardiac dysfunction [7, 8].

In the last 20 years, many randomized clinical trials (RCT) have aimed to determine the characteristics of the best SBT to identify readiness for extubation [9–11]. Two recent RCTs showed that, compared to non-assisted SBTs with a T-piece, low levels of assistance during SBT (8 cmH_2_O pressure-support ventilation (PSV)) yielded a higher rate of successful extubation in patients with simple weaning and in shorter weaning in difficult-to-wean patients [12, 13]. Nevertheless, despite the evidence, approaches to SBT around the world vary widely, from non-assisted to high levels of assistance (e.g., PSV and positive end-expiratory pressure (PEEP)) [14]. Very few studies have focused on the extubation maneuver in the ICU. Two clinical trials by Andreu et al. [15, 16] demonstrated that using 15/10 cmH_2_O positive pressure during cuff deflation and extubation resulted in clinical outcomes similar to those obtained using continuous suction during extubation. Recently, a systematic review on the use of suctioning and positive-pressure during extubation was unable to recommend either approach over the other, and the authors encouraged more studies to elucidate this question [17].

Ultrasonography is a noninvasive diagnostic and monitoring tool that enables assessment of the respiratory muscles and lung aeration [18, 19]. Lung ultrasonography scores (LUS) stratify lung aeration for six regions of each lung; low scores indicate normal aeration, and high scores indicate collapsed areas. Recently, a shorter, more reproducible LUS exploring just four areas of each lung was proposed [20]. High LUS after extubation are associated with extubation failure [20, 21]. Very little is known about decreases in lung aeration during SBTs or extubation. Ultrasonography can also assess the diaphragm and accessory muscles [19]. Diaphragm atrophy and weakness are associated with longer duration of MV and longer weaning [22]. Some preliminary data suggest that during MV oxygenation and lung aeration [23].

### Objectives {7}

The main objective of this study is to determine whether a weaning strategy that aims to preserve lung volume in mechanically ventilated patients ready to wean from the ventilator achieves a higher rate of successful extubation than a weaning strategy based on continuous suction without PEEP during extubation. Secondary objectives were to compare the reintubation rate, ICU and hospital stays, and the loss volume of aerated lung during weaning measured by US between the two approaches. We present the study protocol according to SPIRIT guidelines [24].

### Trial design {8}

Multicenter superiority randomized clinical trial. Patients ready to wean from the ventilator will be randomized to two weaning strategies intended to have different levels of lung volume preservation: control group (30-min SBT using PSV 8 without PEEP and extubation with continuous suction) and experimental group (30-min SBT using PSV 8 + PEEP 5 and extubation without suction).

## Methods: participants, interventions, and outcomes

### Study setting {9}

Any ICU that provides MV could participate in the study. All Spanish ICUs were invited to participate in the study through the collaborative networks of the national scientific societies in Spain (SOCMIC, SEMICYUC). Finally, 29 ICUs agreed to collaborate; 4 of these will use LUS to measure lung aeration and muscular thickness during weaning. Appendix 1 provides additional information on participating centers.

To participate in the ultrasound outcome, centers must have adequate equipment and expertise in LUS according to the APECHO study [25]. The coordinating center will review at least five scans per center to ensure that they meet the required conditions.

### Eligibility criteria {10}

Inclusion criteria:

1. Patients aged ≥ 18 years undergoing MV for ≥ 24 h
2. Patients meeting weaning criteria according to Bole et al. [2]: adequate cough (ability to raise secretions up the endotracheal tube), absence of excessive secretions (< 3 suctions in the last 8 h), resolution or improvement of the pathology that motivated intubation, clinical stability (heart rate (HR) < 140 bpm, systolic blood pressure (BP) 90–160 mmHg without vasopressors or at minimum doses), adequate oxygenation (SatO_2_ > 90% with FiO_2_ < 0.4), adequate pulmonary function (respiratory rate (RR) < 35 breaths per minute, maximal inspiratory pressure <  − 20 cmH_2_O, tidal volume (Vt) > 5 ml/kg, current volume (VC) > 10 ml/kg, RR/Vt < 100 rpm/l, no significant acidosis), adequate mentation (no sedation or adequate mentation on sedation, Glasgow Coma Scale (GCS) > 13)

General exclusion criteria:

1. Tracheostomy
2. Do-not-resuscitate orders
3. Preference for a specific weaning strategy by the physician in charge
4. Absence of informed consent
5. Mental incapacity without legal representation

Specific exclusion criteria for ultrasound:

1. Inadequate ultrasound window (subcutaneous emphysema, lung bullae, large or thick bandages, pneumothorax, etc.)
2. Previous neuromuscular disease that may affect diaphragm and muscular function
3. Absence of qualified staff for the ultrasound assessment at the time of enrollment

### Who will obtain informed consent? {26a}

The clinical team (or research coordinating staff if available) at the participating centers will determine whether patients meeting weaning criteria are eligible [2]. Once the patient is considered eligible, one member of the investigating team (coordinator, nurse, physician) will inform the patient if he/she is able to communicate or the patient’s relatives as a substitute decision maker (SDM) about the study procedure and will invite them to participate. As each treatment arm can be considered the standard of care and as the decision to perform an SBT cannot be postponed, consent can be obtained from SDMs by telephone. Before any patient’s data can be included in the study database, the patient or his/her SDM must have signed the informed consent form. If consent cannot be obtained, none of the data will be included in the analysis of the outcomes, but the patient will be included in the study flowchart.

### Additional consent provisions for collection and use of participant data and biological specimens {26b}

N/A. No biological specimens in ancillary studies are planned.

## Interventions

### Explanation of the choice of comparators {6b}

Both groups of randomized patients will undergo an SBT and extubation maneuver with different levels of lung volume preservation. Patients allocated on the intervention group will do an SBT using PSV 8 cmH_2_O PEEP 5 cmH_2_O, and the extubation maneuver will be without suction. The comparator in control group will be an SBT using PSV 8 cmH_2_O with no PEEP, what recently showed better successful extubation rates [12, 13] and extubation with continuous suction.

### Description of the intervention {11a}

Before randomization, attending physicians must state whether they intend to apply prophylactic post-extubation high-flow nasal cannula (HFNC) or noninvasive MV, respiratory therapy, or reconnection to clinical settings for rest before extubation.

Patients enrolled will be randomized to one of the two strategies of weaning and extubation:

1. Control group: SBT for 30 min using PSV 8 cmH_2_O without PEEP, followed by extubation with continuous suctioning
2. Intervention (lung-volume preservation) group: SBT for 30 min using PSV 8 cmH_2_O with PEEP 5 cmH_2_O (total inspiratory pressure 8 cmH_2_O), followed by extubation with positive pressure without suctioning

#### SBT procedure

It is recommended but not mandatory to perform two inspiratory occlusions of the ventilator between enrollment and the SBT to determine:

- The airway occlusion pressure (P0.1), as a surrogate of respiratory drive; P0.1 is the negative pressure generated by the patient in the first 100 ms of an occluded inspiration
- Occlusion pressure (Pocc), to estimate inspiratory effort; it is the negative pressure generated by the patient during a single inspiratory occlusion
- Maximal inspiratory pressure (aka, negative inspiratory force) measured immediately after a 20-s inspiratory occlusion so that the patient generates the maximal inspiratory effort

Patients will be in Fowler’s position (seated at 45°), tracheal secretions will be suctioned before SBT, and FiO_2_ will remain at the same level as during MV. In high-risk patients, a cuff-leak test to detect laryngeal obstruction is recommended before the SBT.

During SBTs, if any of the SBT failure criteria appear, the SBT will be discontinued and the ventilator will be switched back to the previous settings. Further SBTs in these patients will not be randomized and may be performed at the discretion of the attending team.

According to published literature [2] the criteria for SBT failure will include:

1. Subjective indexes: neurological (agitation, anxiety, depressed mental status, delirium), diaphoresis, cyanosis, evidence of increasing respiratory effort (evident accessory muscle activity, facial signs of distress, dyspnea)
2. Objective indexes: PaO_2_ ≤ 60 mmHg or SatO_2_ < 90% on FiO_2_ ≥ 0.5; PaCO_2_ > 50 mmHg or an increase in PaCO_2_ > 8 mmHg; acidosis with pH < 7.32; RR/Vt > 105 breaths per min/L; RR > 35 breaths/min; HR > 140 bpm or an increase of 20%; systolic BP > 180 mmHg or an increase of 20%; systolic BP < 90 mmHg; cardiac arrhythmia

Passing the SBT is defined as completing it without any criteria for failure appearing. When a patient passes an SBT, it is recommended, but not mandatory, to reconnect them to their previous MV settings to rest for 1 h before extubation [5].

#### Extubation procedure

Oral secretions will be suctioned before extubation in both groups.

In the control group, the extubation maneuver will be performed disconnecting the patient from the ventilator, introducing a suction catheter in the tube (approximately 30 cm), deflating the cuff and removing the tube with continuous suctioning. In the intervention group, the extubation maneuver will be performed without disconnecting the patient from the ventilator and maintaining a positive pressure of PEEP 5 cmH_2_O while deflating the cuff and removing the tube.

#### Follow-up after extubation

Extubation failure is defined as meeting ≥ 1 of the following criteria ≤ 72 h after extubation, regardless of whether reintubation is required: respiratory acidosis, pH < 7.32, PaCO_2_ > 45 mmHg; SatO_2_ < 90% or PaO_2_ < 60 mmHg with FiO_2_ ≥ 50%; RR > 35 breaths/min; low level of consciousness (GCS < 13); uncontrollable agitation; or clinical signs of respiratory muscle fatigue.

If extubation failure develops, the patient may receive noninvasive MV, HFNC, or reintubation as the standard of care at the discretion of the attending physician. Reintubated patients will not be randomized in further SBTs, and all further treatments including weaning strategy will be at the discretion of the attending physician.

Survival will be followed to 90 days after enrollment, regardless of where the patient is (ICU, hospital ward, discharged, or dead).

#### Ultrasound monitoring

In patients included in the ultrasound sub-study, the weaning process will be monitored using ultrasound to examine the lungs, diaphragm, and intercostal muscles at different times during the SBT and extubation (see the “Participant timeline {13}” section). Ultrasound will be performed as previously published [18, 19]. At all participating centers, to enable calculation of the loss in diaphragm thickness from admission to weaning to be calculated, all patients on MV will undergo ultrasound of the diaphragm at admission if possible as the standard of care.

To reduce bias, US images will be acquired without measurements. The centers acquiring the images will send them in DICOM format to the coordinating center, and two members of the investigating team (radiologist, intensivist) will measure all parameters and calculate the mLUS and respiratory muscle thicknesses and fraction using the DICOM reader software.

### Criteria for discontinuing or modifying allocated interventions {11b}

Patients with accidental or self-extubation during SBT will be analyzed on an intention-to-treat basis. If the physician in charge considers it necessary to suction a patient before extubation due to excessive secretions, the patient will also be analyzed on an intention-to-treat basis.

If the physician in charge decides to extubate a patient who has met the criteria for SBT failure or not to extubate a patient who has passed, those patients will be analyzed as extubated or not extubated according to their real situation. If there are many such protocol violations, a post hoc analysis will be performed.

### Strategies to improve adherence to interventions {11c}

N/A. As both approaches can be considered standard of care, we do not anticipate any strategy to improve adherence to the protocol.

### Relevant concomitant care permitted or prohibited during the trial {11d}

Any prophylactic treatment applied after SBT, such as reconnection to the ventilator, HFNC, or noninvasive MV, will be at the discretion of the attending care team and local protocols. We encourage deciding on the treatment before randomization; however, the attending team can change the approach and treatment as needed. This information will be collected in the clinical research document.

Although we discourage the use of noninvasive MV for postextubation respiratory failure, the care team can use it if they deem it necessary.

Any other components of standard care, such as physiotherapy, nutrition, hemodynamic management, or antibiotics, will remain at the discretion of the attending physician and local protocols.

### Provisions for post-trial care {30}

N/A. The patients enrolled will not need any special post-trial care other than usual care.

## Outcomes {12}

Primary outcome:

The primary outcome will be the rate of successful extubation at 72 h after the first SBT. *Successful extubation rate* = *(number of patients extubated after the first SBT and not reintubated at 72 h)/(all patients who underwent SBT).*

Secondary outcomes:

- Postextubation respiratory failure and reintubation rate at 72 h after the first SBT. *Postextubation respiratory failure* = *(number of patients extubated after first SBT who develop respiratory failure within 72 h)/(number of patients extubated after first SBT). Reintubation rate* = *(number of patients extubated after the first SBT who are reintubated within 72 h)/(number of patients extubated after the first SBT)*
- ICU and hospital lengths of stay
- ICU, hospital, and 90-day survival rates
- Incidence of tracheostomy
- Lung aeration: difference between mLUS scores recorded before SBT and after extubation

Exploratory outcome:

- Diaphragm and intercostal muscle thickness (mm) and thickening fraction at the beginning and at the end of the SBT. *Thickening Fraction* = *(maximal thickness – minimal thickness)/minimal thickness (%)*
- Patterns of mLUS and changes in diaphragmatic and intercostal muscles in patients who fail weaning
- Changes in lung aeration in the posterior-basal regions of the lung during SBT and extubation
- Diaphragmatic function in relation to maximal inspiratory pressure, P0.1, and Pocc

### Participant timeline {13}

Tables 1 and 2 summarize the data collection schedule. We will collect information on outcomes at each stage of recruitment, randomization, treatment allocation, follow-up, and analysis to report patient flow according to CONSORT guidelines [26]. We will record the number of patients who meet exclusion criteria, the number of patients who qualified for inclusion but who were not willing to participate, the number of patients assigned to the intervention arm, the number of patients assigned to the control arm, the number of patients for whom follow-up data was available, the number of patients included in the analyses, and the number of withdrawals.

**Table 1:** Summary of procedures according to the timeline

	Before weaning	Before SBT	During SBT	Extubation	After extubation
Clinical	Daily screening of patients meeting weaning criteria Determine prophylactic treatment before randomization: none, reconnection to clinical settings, HFNC, NIV, physiotherapy	Ventilator settings, vital signs ^a^Assessment of P0.1, Pocc, MIP	Determine extubation criteria at 30 min of SBT Determine deviations of protocol in extubation decision Subjective dyspnea^a^ Self-confidence in breathing^a^ Settings and vital signs at the beginning and at the end of SBT If available as SOC^a^: blood gas	Prophylactic treatment applied: rest on clinical setting, NIV, HFNC	Outcome at 72 h: - Postextubation respiratory failure - Reintubation ICU and hospital LOS Survival at 90 days
Ultrasound	First 48 h of MV^a^: diaphragm thickness (mm)	1. ^a^Diaphragm thickness and TF during MIP and Pocc maneuvers 2. At clinical settings: mLUS, diaphragm and intercostals thickness and TF	1. Successful SBT (at 30 min): mLUS, diaphragm^a^ and intercostals thickness and TF^a^ 2. Failed SBT: (before reconnecting to clinical settings): mLUS, diaphragm^a^ and intercostal thickness and TF^a^	As soon as possible after extubation (< 1 h): mLUS, diaphragm^a^ and intercostals thickness and TF^a^	At 24 h of extubation^a^: repeat mLUS, diaphragm and intercostal thickness and TF

*HFNC* high-flow nasal cannula; *NIV* non-invasive ventilation, *Pocc* occlusion pressure in one single breath, *MIP* maximal inspiratory pressure; *SBT* spontaneous breathing trial; *TF* thickening fraction; *mLUS* modified lung ultrasound score

^a^Optional

**Table 2:** Summary of measures and variables according to the timeline

	Before SBT	During SBT	After SBT	Extubation	After extubation	72 h after SBT	ICU discharge	Hospital discharge	90-days follow up
Screening eligible patients	x								
Informed consent	x								
Clinical assessment: HR, RR, BP, SaO_2_	x	x	x	x					
Ventilator setting	x	x	x						
Blood gas			x		x	x			
Lung ultrasound (mLUS)	x	x	x	x	x				
Diaphragm and intercostal thickness and thickening fraction	x	x	x						
HFNC settings				x	x	x			
NIV settings				x	x	x			
Respiratory failure					x	x			
Reintubation					x	x			
Tracheostomy					x	x	x	x	x
Survival							x	x	x

*SBT* spontaneous breathing trial; *HR* heart rate; *RR* respiratory rate; *BP* blood pressure; *SaO*_*2*_ arterial saturation of oxygen; *mLUS* modified lung ultrasound score; *HFNC* high-flow nasal cannula; *NIV* non-invasive ventilation

### Sample size {14}

According to our previous results [12], the successful extubation rate expected in the control group is 82%. In the intervention group, we hypothesize an absolute increase in the successful extubation rate of 5%. Considering an alpha error of 0.05 and a power of 80%, 822 patients in each group are required to confirm the hypothesis. Recruitment started in January 2023 in 29 ICUs in Spain. The estimated date for reaching the required sample size is December 2024.

Tenza et al. [20] report that a difference in lung aeration calculated by mLUS (range, 0–24 points) of 1 point (SD 2.19) is clinically significant for weaning failure; thus, assuming an alpha error of 5% and a power of 80%, a total of 186 patients are required (93 in each group) for the lung volume outcome based on mLUS.

### Recruitment {15}

Most patients meeting weaning criteria in the participating ICUs can be enrolled. Given the number of participating sites (29 ICUs), we consider it completely feasible to achieve the sample size within 24 months from the starting date.

## Assignment of interventions: allocation

### Sequence generation {16a}

Center-stratified blocked randomization. Patients will be randomized in a 1:1 ratio to one of the two weaning strategies. A randomization list will be produced by a computer-generated random-number sequence in blocks of a predetermined size to ensure consistent patient distribution in the two groups. The randomization list will be generated by the Clinical Research Unit; control allocation to each group will be performed by REDCap. The researchers will not be aware of the randomization scheme. Neither the investigators nor the attending physicians will be blinded to the study group.

### Concealment mechanism {16b}

The allocation will be performed by REDCap after confirming all inclusion and exclusion criteria.

### Implementation {16c}

The randomization list will be generated by the Clinical Research Unit. Any member of the site investigating team (physician, nurse, research coordinator) will enroll patients. All sites will have a “Randomization profile” in REDCap that allows for the confirmation of inclusion criteria and the randomization of patients to each allocation.

## Assignment of interventions: blinding

### Who will be blinded {17a}

The care team and the investigators will not be blinded to the randomization group as the parameters on the ventilator will differ between each group.

### Procedure for unblinding if needed {17b}

N/A. Unblinded study.

## Data collection and management

### Plans for assessment and collection of outcomes {18a}

Before starting the study, a technical and competence evaluation of the centers participating in the ultrasound substudy will be conducted. At each of these centers, we will designate at least one collaborator who has experience in lung ultrasound according to the recommendations of the APECHO study [25] accredited either through a course endorsed by an intensive care or ultrasound scientific society or through performing at least 25 complete examinations supervised by an expert. This collaborator will supervise the personnel in their unit to ensure the acquisition of quality ultrasound images.

The centers must have suitable convex and linear probes capable of recording 3- to 10-s video clips and be able to download ultrasound images in DICOM format. Moreover, each center’s aptness will be assessed in a trial period where they must send at least 5 scans including all LUS windows as well as images examining diaphragmatic and intercostal thickening from 5 different patients to confirm that the scans meet the specified requirements and can be correctly interpreted by an independent observer.

At the baseline assessment: age, sex, weight, height; comorbidities (heart disease, neurological disease, chronic obstructive pulmonary disease, diabetes, cancer, chronic renal insufficiency, liver disease); diagnosis at admission to the ICU; reason for intubation; APACHE II at admission; date of admission to the hospital and to the ICU; and if possible, diaphragm thickness within 48 h of starting MV.

Before starting SBT: ventilatory mode before SBT, FiO_2_, RR, SaO_2_, Vt, PEEP, HR, BP; ultrasound images of the lungs, diaphragm, and accessory muscles.

During the SBT: date and time that SBT started; FiO_2_, RR, SaO_2_, Vt, HR, BP, subjective dyspnea scale (0–10).

At the end of SBT: failed or successful SBT, extubation date and time, FiO_2_, RR, SaO_2_, Vt, HR, BP, subjective dyspnea scale (0–10), and subjective confidence in breathing after extubation. If blood gas forms part of standard of care: pH, PaO_2_, PaCO_2_, bicarbonate, and base excess. Ultrasound images of the lungs, diaphragm, and accessory muscles.

After a successful SBT: ultrasound images of the lungs, diaphragm, and accessory muscles after extubation and at 24 h. If any prophylactic treatment is applied:

- For reconnection to clinical settings on the ventilator for 1 h before extubation: time on the ventilator, ventilator settings (mode, FiO_2_, RR, Vt, PEEP)
- For noninvasive MV: maximal IPAP and EPAP, highest RR, highest FiO_2_, lowest SaO_2_, date and time of removal
- For HFNC: maximal flow, highest RR, highest FiO_2_, lowest SaO_2_, date and time of removal
- For respiratory physiotherapy: date and time of start and finish

Respiratory failure ≤ 72 h postextubation: date and time of failure; reason for failure. If blood gas analysis is performed after failure: PaO_2_, PaCO_2_, pH, bicarbonate, and base excess. If HFNC is used to treat failure: date and time of start and finish, FiO_2_, maximal flow, highest RR, lowest SaO_2_. If noninvasive MV is used to treat failure: date and time of start and finish, FiO_2_, maximal flow, highest RR, lowest SaO_2_.

Reintubation at 72 h: date and time of reintubation; reason for reintubation. Date and time of definitive removal of MV. Date of tracheostomy if it is performed.

90-day follow-up: ICU discharge date, hospital discharge date. Vital status 90 days after enrollment. In case of death, date of death.

### Plans to promote participant retention and complete follow-up {18b}

All the variables recorded at 90-day follow-up can be easily obtained from patients’ medical records (e.g., date of definitive removal of MV, need for tracheostomy, ICU and hospital discharge dates, and 90-day survival).

### Data management {19}

Data will be recorded in an electronic case report form (eCRF) designed with the REDCap program [27, 28]. The REDCap platform will be hosted on the institution’s servers with the security protocols deemed prudent by the institution. The data will be stored on the local web server where the organization has installed the software. REDCap is published on Althaia’s website https://www.althaia.cat/redcap. To access the application, users need to log in with their email and password. A system has been implemented so that only the application service can send data to the back office via a firewall that only allows requests from the application’s IP addresses. The web server has the HTTP X-Frame-Options header configuration enabled with the value “same-origin” to prevent clickjacking attacks. The principal investigator and the study sponsor, as data owners, will be responsible for data custody. Investigators at each participating center will only be able to record and consult data from their own patients. Only the principal investigator of the study will have access to all patient data in the study.

To prevent errors entering data, a range of valid values will be defined for all variables in the eCRF.

### Confidentiality {27}

To ensure pseudonymization of the data, the data will be collected in a database specifically designed for the study in a dissociated manner. REDCap assigns each patient a numeric code that has no relation to the patient’s personal data. The patient’s demographic, clinical, and laboratory data will be entered into the database associated with this code.

Each center will be responsible for downloading the ultrasound images WITHOUT any measurements in DICOM format. These images will be sent with an identification code to the center coordinating the study using the FileCloud program, which complies with data protection requirements.

### Plans for collection, laboratory evaluation, and storage of biological specimens for genetic or molecular analysis in this trial/future use {33}

NA. No biological or laboratory evaluation will be performed.

## Statistical methods

### Statistical methods for primary and secondary outcomes {20a}

Continuous variables will be summarized as means and standard deviations (if normally distributed) and as medians and interquartile ranges (if non-normally distributed). Categorical variables will be expressed as absolute values and relative frequencies.

We will test for significant differences between the control and intervention groups in the primary, secondary, and exploratory outcomes. To compare continuous variables, we will use Student’s *t*-test if both groups have normal distributions or the Mann–Whitney *U* test otherwise. To compare categorical variables, we will use the *χ*^2^ test or Fisher’s exact test, as appropriate, or bilateral exact *p*-value in contingency tables when the expected frequency is < 5.

In the bivariate analysis, Kaplan–Meier survival curves will be constructed, and the log-rank test will be used to compare them. Crude and adjusted hazard ratios and 95% confidence intervals will be calculated using simple or multivariable Cox proportional regression models. Covariates, that plausibly fit the criteria of confounder on the basis of prior knowledge, will be introduced into the multivariable model. Causal models using directed acyclic graphs (DAGs) will be used to select a minimum set of confounders [29]. The proportionality of hazards will be verified by examining Schoenfeld residual plots.

Outcomes will be analyzed on an intention-to-treat basis. Two-sided *p*-values ≤ 0.05 will be considered statistically significant. Data will be analyzed using IBM SPSS Statistics v.29 (IBM Corporation®, Armonk, New York) and R® v.4.3.2 (R Foundation for Statistical Computing, Vienna, Austria).

### Interim analyses {21b}

When half of the sample has been recruited, an interim analysis will be conducted by external personnel. We will use the O’Brien and Fleming boundaries to determine whether to continue the trial [27]. The threshold *p*-value for the interim analysis will be set at 0.0054. If significant differences (*p* < 0.0054) are found between the study groups, the inclusion of patients will be stopped.

### Methods for additional analyses (e.g., subgroup analyses) {20b}

A random-effects multilevel logistic regression model will be used to determine variables associated with 72-h successful extubation, taking into account the effect of the participating hospital. Patient characteristics that are associated with 72-h successful extubation in the bivariable analysis will be introduced in the random-effects multilevel logistic regression model as first-level variables and hospital as a second-level variable. Odds ratios (ORs) and median ORs with 95% confidence intervals will be used to measure the association between each covariate and 72-h successful extubation. Post hoc analyses will be performed for primary, secondary, exploratory, and post hoc outcomes among subgroups defined by baseline demographic characteristics. Effect sizes will be evaluated by computing absolute risk differences with 95% confidence intervals for binary outcomes and differences in means with 95% confidence intervals for continuous outcomes.

### Analytical methods to handle non-adherence to protocol and statistical methods to handle missing data {20c}

The study will start with patients on MV > 24 h who meet the criteria for weaning [2] All patients with any exclusion criterion, including the decision of the treating physician, will be excluded before randomization. The primary and secondary outcomes will be based only on randomized patients.

All patients will be analyzed in the group to which they are randomized according to the intention-to-treat principle, with no exclusion after randomization. Patients extubated outside of protocol will be analyzed as failed SBT. The reintubation rate will be recorded only among patients who complete the SBT.

Missing data will be imputed (median values for continuous variables and mode values for categorical variables) for independent variables with less than 5% missing data.

### Plans to give access to the full protocol, participant-level data, and statistical code {31c}

This trial has been registered on ClinicalTrials.gov, and the full protocol can be accessed there.

The dataset and statistical code of this study will be available for specific proposals through correspondence with the investigators.

## Oversight and monitoring

### Composition of the coordinating center and trial steering committee {5d}

Principal investigator and study coordinator:

- Design and conduct of the study
- Preparation of the protocol and revision
- Submission of the protocol and related documents to ClinicalTrials.gov and REB
- Supervision of data entry and providing updates about enrollment through monthly newsletters
- Integration of the data from all sites in a single database

Site investigators and research team:

Conducting the study at each participating center (screening, enrollment, randomization, data collection).

### Composition of the data monitoring committee, its role and reporting structure {21a}

This study has no data monitoring committee. The principal investigator and study coordinator will review data entry weekly and will update the participating centers about the rate of enrollment.

### Adverse event reporting and harms {22}

As both groups can be considered standard of care, we do not anticipate any adverse events other than those related to weaning from MV and extubation.

### Frequency and plans for auditing trial conduct {23}

This study has no data monitoring committee. To ensure that all centers are complying with the protocol, the principal investigator and study coordinator will maintain close contact with collaborators at each center and will review data entry weekly. The sponsor is not involved in any aspect of the study.

### Plans for communicating important protocol amendments to relevant parties (e.g., trial participants, ethical committees) {25}

Any modifications to the protocol that may impact the conduct of the study, the potential benefit of the patient, or patient safety, including changes in study objectives, design, patient population, sample size (except the changes after the interim analysis as described above), procedures or intervention, or significant administrative aspects, will require a formal amendment to the protocol. Such amendment will first be discussed at investigator meetings, be agreed upon by the steering committee and the principal investigator, and finally approved by the Institutional Review Board/Ethics Committee prior to implementation. The health authorities will be notified in accordance with local regulations.

### Dissemination plans {31a}

Regardless of the results of this trial, we expect to present them at international conferences and to publish them as original papers in peer-reviewed scientific journals.

## Discussion

To the best of our knowledge, this is the first RCT that aims to compare two weaning strategies that likely result in different levels of lung aeration throughout the weaning process (SBT and extubation maneuver).

The reintubation rate has remained stable (10–20%) for the last 15 years. Although this percentage might seem low, patients who require reintubation are more likely to die, and weaning failure has a large impact on health and healthcare costs [6, 8]. Thus, it remains important to investigate weaning strategies that might help reduce the likelihood of extubation failure.

Currently, the best SBT for extubation is not well defined in guidelines [30, 31]: there is still a wide variety of SBTs with different levels of pressure [14]. Previous trials were underpowered to demonstrate that positive pressure than yields a higher rate of successful extubation than continuous suction [15]. We did not aim to reproduce the earlier RCTs with a larger sample; rather, we decided to compare weaning strategies likely to result in different levels of lung collapse not only during extubation but also during the SBT. Our primary outcome is successful extubation, because it is the most directly related outcome for weaning. Although some authors have used longer periods (up to 7 days) to define successful extubation [32], we decided to use a shorter period (72 h) that is also common in the literature, because reintubation after this time is unlikely to be related to weaning practice.

Including an additional ultrasound outcome in this RCT is intended to provide information complementary to that derived in testing the clinical hypothesis. We do not aim to promote the universal use of ultrasound to monitor the lungs, diaphragm, and intercostal muscles throughout weaning in all patients, as this would not be feasible in daily practice. However, if we find that lung collapse is a major cause of weaning failure, ultrasound may help clinicians make the right decision about extubation in selected patients (i.e., high risk and clinically borderline).

Even if our study demonstrates the superiority of the lung-volume preservation approach, it will be unable to identify whether PEEP-SBT or positive pressure extubation is more important to preserve lung volume. As the successful extubation rate has remained stable in recent decades, any strategy attempting to improve this rate will require a large population. Our estimated sample size (1600 patients) may seem difficult to reach. However, 29 hospitals are already enrolling patients at a mean rate of 90 patients/month. So, we believe it is completely feasible to reach the target size in the next 18–24 months.

Recently, some investigators have suggested less stringent weaning criteria, with higher FiO_2_ and PEEP to enable earlier separation of the patient from the ventilator [33]. However, there is not yet enough evidence to support this strategy, and we decided to use the classical weaning criteria that requires lower levels of FiO_2_ and PEEP. In this RCT, an SBT is not indicated unless the patient is clinically ready to be extubated; for this reason, the protocol calls for patients who pass the SBT to be extubated [34]. However, we anticipate some deviations from the protocol; some patients might be extubated after a failed SBT or conversely some patients might not be extubated after a successful SBT. These deviations can occur for many reasons (clinician decision, accidental extubation, etc.), and they will be collected in the eCRF for post hoc analysis. However, for the main analysis, these patients will be considered extubated or not extubated according to their real situation.

Our study protocol has some limitations. First, as in any other weaning study, the investigators cannot be blinded to the weaning strategy. Second, as explained above, we anticipate some deviations from protocol; a sensitivity analysis will be performed to determine the bias related to these deviation. Third, the design of the study will not elucidate the specific effects of PEEP during SBT and of positive pressure during extubation; thus, the result must be interpreted as the synergy of the two factors. Fourth, any prophylactic treatment after extubation aiming to reduce reintubation will not be controlled or standardized. However, we anticipate a similar use in both randomization groups, and we will perform a sensitivity analysis to determine the effects, if any, of each treatment (HFNC, noninvasive MV, resting on clinical settings, or physiotherapy). Fifth, although we recommend against noninvasive MV or HFNC to treat respiratory failure after extubation, we allow this possibility as previous results have shown that these approaches are widely used, and some authors still encourage their use [35].

## Trial status

Protocol version 4. May 2023.

At the time of submission, the enrollment of patients has already started at the 29 participating sites.

### Supplementary Information

Additional file 1.

## Data Availability

Only the study promotor and principal investigator will have access to the final dataset. The dataset and statistical code of this study will be available for specific proposals through correspondence with the investigators.

## Abbreviations

BP: Blood pressure
eCRF: Electronic case report form
HR: Heart rate
ICU: Intensive care unit
mLUS: Modified lung ultrasound score
MV: Mechanical ventilation
PEEP: Positive end-expiratory pressure
Pocc: Occlusion pressure
PSV: Pressure support ventilation
SDM: Substitute decision maker
SBT: Spontaneous breathing trial
