# Supplementary material for: Effect of lung volume preservation during spontaneous breathing trial on successful extubation in patients receiving mechanical ventilation: protocol for a multicenter clinical trial
Source: Trials. 2024 Jul 16;25:481. doi: 10.1186/s13063-024-08297-1 (PMC11251308; doi:10.1186/s13063-024-08297-1)
Supplement: Supplementary file 1 — Additional file 1. [file 13063_2024_8297_MOESM1_ESM.docx]

**Appendix 1.** List of participating centers and site investigators.

1. Althaia Xarxa Assistencial de Manresa. Manresa, Spain. Coordinator Center. Investigator team: Gina Rognoni, Herbert Baquerizo, Montserrat Batlle, Sílvia Cano, Sílvia Garcia-Castrillón, Xavier Andorrà, Yenifher Tua, Anna Arnau. Study Coordinator: Rafael Fernández. This center participates in the ultrasound substudy.
2. Hospital de la Santa Creu i Sant Pau. Principal Investigator: Carles Subirà. (This center is not enrolling patients).
3. Hospital Universitario de Canarias. Tenerife, Spain. Investigator team: Carolina García, Claudia Domínguez-Curell.
4. Txagorritxu Hospital Universitario Araba. Gasteiz, Spain. Investigator team: Sara Cabañes, Ana Villagrá.
5. Hospital de Mataró. Mataró, Spain. Investigator team: María de la Torre, Juan Arturo Méndez. This center participates in the ultrasound substudy.
6. Hospital Clínico Universitario de València. València, Spain. Investigator team: Beatriz Quevedo.
7. Hospital General de Granollers. Granollers, Spain. Investigator team: Cristina Pedrós. This center participates in the ultrasound substudy.
8. Complexo Hospitalario Universitario de Ourense. Ourense, Spain. Investigator team: Ana Tizón, Lorena del Río Carbajo. This center participates in the ultrasound substudy.
9. Hospital Universitari de Tarragona Joan XXIII. Tarragona, Spain. Investigator team: Naralia Murillo, Laura Claverías, Sara Manrique.
10. Hospital Universitario de Henares. Coslada, Spain. Investigator team: Laura Parro, Federico Gordo.
11. Hospital Universitario de Pontevedra. Pontevedra, Spain. Investigator team: Fernando Erias, Ana Ortega.
12. Hospital Son Llàtzer. Palma de Mallorca, Spain. Investigator team: Gemma Rialp.
13. Hospital General Universitario de Castelló. Castelló de la Plana, Spain. Investigator team: Susana Altaba.
14. Hospital Universitario Marqués de Valdecilla. Santander, Spain. Investigator team: Alejandro González-Castro.
15. Hospital Universitari de la Vall d’Hebron. Barcelona, Spain. Investigator team: Andrés F. Pacheco, César Laborda.
16. Hospital General Universitario Santa Lucía. Cartagena, Spain. Investigator team: Pablo Bayoumi.
17. Hospital General Universitario de Elche. Elx, Spain. Investigator team: Norma Medrano, Elena Tenza.
18. Hospital Universitari Sant Joan de Reus. Reus, Spain. Investigator team: Imma Vallverdú.
19. Hospital General Universitario Morales Messeguer. Murcia, Spain. Investigator team: Áurea Higón, Laura López.
20. Hospital Arnau de Vilanova. València, Spain. Investigator team: María D. Navarro.
21. Hospital Universitari Mútua de Terrassa. Terrassa, Spain. Investigator team: María del Mar Ferández, Alirio Falcón.
22. Hospital Universitario de la Princesa. Madrid, Spain. Investigator team: Elena Keough.
23. Hospital de Sagunto. Sagunt, Spain. Investigator team: David Arizo.
24. Hospital Regional Universitario de Málaga. Málaga, Spain. Investigator team: Juan F. Martínez.
25. Hospital Sagrat Cor. Barcelona, Spain. Investigator team: Núria Durán.
26. Hospital Universitario Central de Asturias. Oviedo, Spain. Investigator team: Raquel Rodríguez.
27. Hospital de Sant Joan Despí Moisès Broggi. Sant Joan Despí, Spain. Investigator team: Melinda R. Popoviciu-Koborzan.
28. Hospital Virgen de las Nieves. Granada, Spain. Investigator team: Isabel Guerrero, José Miguel Pérez.
29. Hospital Verge de la Cinta. Tortosa, Spain. Investigator team: Pablo Concha, Ferran Roche.
30. Complejo Hospitalario Universitario de Santiago. Santiago de Compostela, Spain. Investigator team: Patricia Barral.
